# Supplementary material for: Anisotropic photoemission time delays close to a Fano resonance
Source: Nat Commun. 2018 Mar 6;9:955. doi: 10.1038/s41467-018-03009-1 (PMC5840338; doi:10.1038/s41467-018-03009-1)
Supplement: Supplementary file 1 — Supplementary Information [file 41467_2018_3009_MOESM1_ESM.pdf]

Supplementary information

Supplementary Note 1: Details of the theoretical model used to calculate angle dependent atomic delays close to a Fano resonance

The theoretical curves shown in Fig. 5 in the main text have been computed according the following approach.

The angularly resolved $2n$-th SB amplitude is given by

$$A_{\mathrm{SB}}^{2n}(\hat{\Omega}) = \sum_m \left| A_{m,f\leftarrow g}^{2n+1}(\hat{\Omega}) + A_{m,f\leftarrow g}^{2n-1}(\hat{\Omega}) \right|^2, \qquad (1)$$

where m is the magnetic quantum number of the parent-ion and $A_{m,f\leftarrow g}^{2n-1}$ ($A_{m,f\leftarrow g}^{2n+1}$) is the amplitude representing the absorption of the ($2n$-1)-th [($2n$+1)-th] harmonic from the ground state g followed by the absorption (emission) of an IR photon

$$A_{m,f\leftarrow g}^{2n\pm1}(\hat{\Omega}) = F_{\mathrm{XUV,IR}}^{\pm}(\tau) e^{\pm i\omega_{IR}\tau} c_m^{\mp}(\hat{\Omega}),$$

$$c_m^{\mp}(\hat{\Omega}) = \sum_{l=1,3} Y_{lm}(\hat{\Omega}) e^{i\Theta_l(E)} M_{lm,f\leftarrow g}^{2n\pm1}(E_g + 2n\omega_{\mathrm{IR}}). \qquad (2)$$

Here, $Y_{lm}(\hat{\Omega})$ is the spherical harmonic defining the photoelectron direction ($\hat{\Omega}$), $\Theta_l(E)$ is the phase associated to the final scattering state, $\Theta_l(E) = \sigma_l(E) + \delta_l(E) - \frac{l\pi}{2}$, with $\sigma_l(E)$ the Coulomb phase shift and $\delta_l(E)$ the short-range phase shift, and $M_{lm,f\leftarrow g}^{2n\pm1}(E_g + 2n\omega_{\mathrm{IR}})$ is the two-photon transition matrix element. Notice that l runs over 1 and 3, since the escaping electron can only be described by p or f waves. The factor $F_{\mathrm{XUV,IR}}^{\pm}(\tau)$ contains the pulse

characteristics; we have assumed that both the IR and XUV train have a Gaussian envelope[1].

In the region where the harmonics do not hit any resonance, the two-photon transition matrix elements $M_{lm,f\leftarrow g}^{2n\pm1}$ have been evaluated by using second-order time dependent perturbation theory. The many-body treatment is done within the framework of the random phase approximation with exchange (RPAE), but excluding resonant contributions[2, 3]. We have used this method to evaluate the photoelectron spectrum in the vicinity of the sideband of order 14 (SB14) and to obtain the non-resonant contribution to the photoelectron spectrum in the vicinity of the sideband of order 16 (SB16).

In the region where the harmonics hit the $3s^{-1}5p$ resonance, we have used the two-photon finite-pulse model developed in [1, 4]. In this model, the two-photon transition amplitudes are split into resonant (R) and non-resonant (D) components

$$M_{m,f\leftarrow g}^{2n\pm1} = M_{m,f\leftarrow D\leftarrow g}^{2n\pm1} + M_{m,f\leftarrow R\leftarrow g}^{2n\pm1}, \qquad (3)$$

given by

$$M_{m,f\leftarrow D\leftarrow g}^{2n\pm1} = O_{E_fE}^{D} O_{Eg}^{D} w\left(z_f^{\mp}\right),$$

$$M_{m,f\leftarrow R\leftarrow g}^{2n\pm1} = O_{E_fE}^{R} O_{Eg}^{R} \left[w\left(z_f^{\mp}\right) + (\beta_{Ea} - \epsilon_{Ea}^{-1})(q-i) w\left(z_a^{\mp}\right)\right]. \quad (4)$$

In these expressions, the special functions $w\left(z_f^{\mp}\right)$ and $w\left(z_a^{\mp}\right)$ incorporate the effect of using pulses of finite duration, q is the resonance Fano profile parameter, $\epsilon_{Ea} = 2\left(E_g + 2n\omega_{\mathrm{IR}} - E_r\right)/\Gamma$ is the reduced energy around the resonance, where E_r is the

resonance position and Γ the corresponding autoionization width, and β_{Ea} measures the relative strength of the transition from the intermediate resonance to the final continuum state. All resonance parameters have been taken from existing photoionization experiments[5, 6]. O^D_{Eg} and O^R_{Eg} are the dipole transition matrix elements that couple the ground state to the non-resonant and the resonant intermediate states, respectively. These matrix elements have been taken from earlier *ab initio* calculations[6]. $O^D_{E_fE}$ and $O^R_{E_fE}$ are the dipole transition matrix elements between the intermediate and final continuum states. They have been computed by approximating the radial parts of the continuum orbitals to spherical plane-waves and evaluating the result in the on-shell region[1]. As shown in [1, 4] by explicit comparison with nearly exact solutions of the time-dependent Schrödinger equation, this is a good approximation in energy regions not too close to the ionization threshold. We have used this method to obtain the photoelectron spectrum in the vicinity of SB16.

In both resonant and non-resonant regions, the angularly resolved atomic phase is given by:

$$\Delta\phi_{\text{atomic}} = \tan^{-1}\frac{2|c_1^+||c_1^-|\sin(\Delta\phi_1)+|c_0^+||c_0^-|\sin(\Delta\phi_0)}{2|c_1^+||c_1^-|\cos(\Delta\phi_1)+|c_0^+||c_0^-|\cos(\Delta\phi_0)} \quad (5)$$

$$\Delta\phi_i = \arg\frac{c_i^+}{c_i^-}$$

where the $\hat{\Omega}$ dependences have been dropped for the sake of simplicity.

Supplementary Note 2: Angular dependence of the continuum-continuum (cc) phases

The theory of attosecond delays in laser-assisted photoionization has previously been interpreted using the so-called asymptotic approximation, where atomic phase effects from laser-driven continuum transitions have been estimated using Wentzel–Kramers–Brillouin (WKB) wavefunctions[7]. Within this approximation the phase induced by the laser field, referred to as the continuum-continuum (cc) phase, is independent of the angular momentum of the photoelectron and the processes for absorption (A) and emission (E) of a laser photon are related by $\varphi_{\mathrm{cc}}^{\mathrm{A}} \approx -\varphi_{\mathrm{cc}}^{\mathrm{E}}$. In the case of a single intermediate angular momentum state, such as the *p*-wave from the 1s ground state of hydrogen, the asymptotic approximation implies that the atomic delay should not depend on the angle of photoelectron emission. While the asymptotic approximation describes well the cc-phases at high kinetic energies, exact calculations for hydrogen show angular-momentum dependent cc-phases at low kinetic energies (see Fig. 3 in [7]). Indeed, angle-resolved atomic delay measurements in helium, where there is also one intermediate angular momentum state, have evidenced this breakdown of the asymptotic approximation by measurement of a steep negative atomic delay at large angles relative to the polarization axis of the fields[8].

In argon the interpretation of angle-dependent atomic delay is more complicated because there are two intermediate states: an *s*-wave and a *d*-wave. In Supplementary Figure 1 we present cc-phases extracted from two-photon matrix elements, computed for argon using the method described in[2], by subtracting the one-photon dipole phase corresponding to absorption of one harmonic photon (see Eq. (25) in [7]).

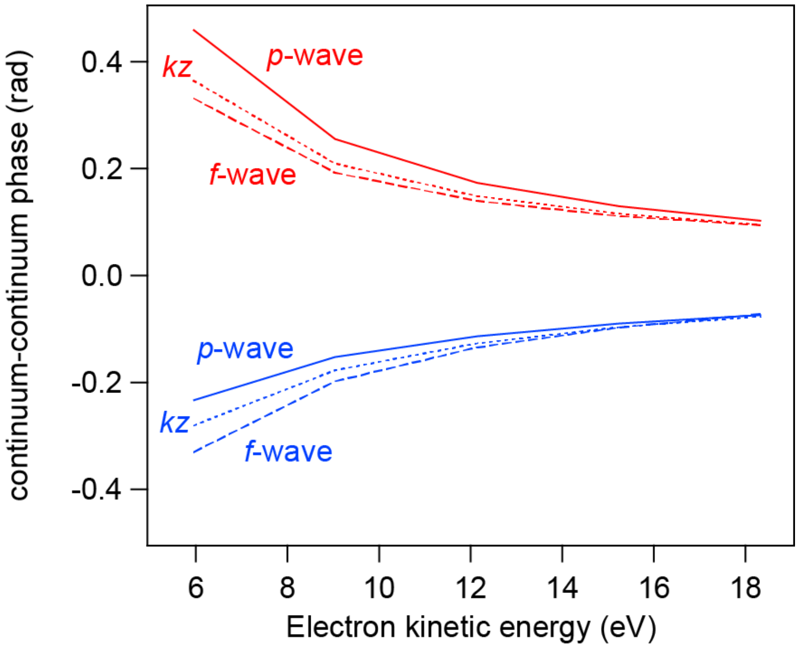

Supplementary Figure 1: Continuum-continuum phases for argon. Theoretical continuum-continuum phases for absorption (upper red) and emission (lower blue) of an IR photon computed using two-photon matrix elements in argon. The curves show the phases calculated for the direction along the polarization axis (dotted lines) and for two different final angular momentum states, *p*-wave (solid lines) and *f*-wave (dashed lines).

The final *f*-wave with m = -1,0,1 has only one intermediate angular momentum state (*d*-wave) that can be easily removed by subtracting the phase of the associated one-photon dipole matrix element to the *d*-wave. In contrast, the final *p*-wave with m=0 has two possible intermediate angular momentum states, which makes the definition of the intermediate dipole phase ill-defined. Fortunately, the *p*-wave with m=1 has only one intermediate angular momentum state (again the *d*-wave) so that the cc-phases can be extracted by subtracting the well-defined one-photon *d*-wave dipole phase.

We find that the argon results are in remarkable agreement with the exact hydrogen calculations presented in [7]: (i) the magnitude of the cc-phases are slightly larger for absorption than for emission and (ii) the cc-phases are decreased (increased) slightly with increased (decreased) final angular momentum close to the threshold.

For comparison we also show the cc-phases in argon for photoelectron emission along the polarization axis $\hat{z}$ by subtracting the associated phase of angle-resolved one-photon dipole matrix element with m=0. As expected, the angle-resolved emission phases lies in between those of the two final partial wave states and at high kinetic energies all cc-phases merge to their respective absorption and emission branches in agreement with the asymptotic approximation.

Supplementary References

[1] A. Jiménez-Galan, F. Martín, L. Argenti, "Two-photon finite-pulse model for resonant transitions in attosecond experiments," *Phys. Rev. A*, **93**, p. 023429, 2016.

[2] J. M. Dahlström, and E. Lindroth, "Study of attosecond delays using perturbation diagrams and exterior complex scaling," *J. Phys. B: At. Mol. Opt. Phys.*, **47**, p. 124012, 2014.

[3] J. M. Dahlström, T. Carette, and E. Lindroth, "Diagrammatic approach to attosecond delays in photoionization," *Phys. Rev. A*, **86**, p. 061402(R), 2012.

[4] A. Jiménez-Galan, L. Argenti, F. Martín, "Modulation of Attosecond Beating in Resonant Two-Photon Ionization," *Phys. Rev. Lett.*, **113**, p. 263001, 2014.

[5] M. Kotur, D. Guenot, A. Jimenez-Galan, D. Kroon , E.W. Larsen, M. Louisy, S. Bengtsson, M. Miranda, J. Mauritsson, C.L. Arnold, S.E. Canton, M. Gisselbrecht, T. Carette, J.M. Dahlström, E. Lindroth, A. Maquet, L. Argenti, F. Martın, and A. L'Huillier, "Spectral phase measurement of a Fano resonance using tunable attosecond pulses," *Nature Comm.*, **7**, p. 10566, 2015.

[6] T. Carette, J. M. Dahlström, L. Argenti, and E. Lindroth, "Multiconfigurational Hartree-Fock close-coupling ansatz: Application to the argon photoionization cross section and delays," *Phys. Rev. A*, **87**, p. 023420, 2013.

[7] J.M. Dahlström, D. Guénot, K. Klünder, M. Gisselbrecht, J. Mauritsson, A. L'Huillier, A. Maquet, and R. Taïeb, "Theory of attosecond delays in laser-assisted photoionization," *Chem. Phys.*, **414**, pp. 53-64, 2013.

[8] S. Heuser, Á. Jiménez-Galán, C. Cirelli, C. Marante, M. Sabbar, R. Boge, M. Lucchini, L. Gallmann, I. Ivanov, A. S. Kheifets, J. M. Dahlström, E. Lindroth, L. Argenti, F. Martín and U. Keller, "Angular dependence of photoemission time delay in helium," *Phys. Rev. A*, **94**, p. 063409, 2016.
